# Purification and In Vitro Evaluation of an Anti-HER2 Affibody-Monomethyl Auristatin E Conjugate in HER2-Positive Cancer Cells

**DOI:** 10.3390/biology10080758

**Published:** 2021-08-07

**Authors:** Isabella Damiani, Silvia Castiglioni, Alicja Sochaj-Gregorczyk, Fabrizia Bonacina, Irma Colombo, Valentina Rusconi, Jacek Otlewski, Alberto Corsini, Stefano Bellosta

**Affiliations:** 1 Department of Pharmacological and Biomolecular Sciences, Università degli Studi di Milano, 20133 Milan, Italy; isabella.damiani@unimi.it (I.D.); silvia.castiglioni@unimi.it (S.C.); fabrizia.bonacina@unimi.it (F.B.); irma.colombo@unimi.it (I.C.); valentina.rusconi2@studenti.unimi.it (V.R.); alberto.corsini@unimi.it (A.C.); 2 Microbiology Department, Faculty of Biochemistry, Biophysics and Biotechnology, Jagiellonian University, Gronostajowa 7, 30387 Krakow, Poland; alicja.sochaj-gregorczyk@uj.edu.pl; 3 Department of Protein Engineering, Faculty of Biotechnology, University of Wroclaw, 50137 Wroclaw, Poland; jacek.otlewski@uwr.edu.pl; 4 IRCCS MultiMedica, Sesto San Giovanni, 20099 Milan, Italy

**Keywords:** affibody, breast cancer, HER2 overexpression, target therapy

## Abstract

**Simple Summary:**

Antibody-drug conjugates (ADCs) represent an innovative class of anticancer agents specifically aimed at targeting cancer cells, reducing damage to healthy tissues but showing some weaknesses. A promising approach for the development of high-affinity tumor targeting ADCs is the use of engineered protein drugs, such as affibody molecules. Our aim was to develop a more efficient purification method for the cytotoxic conjugate Z_HER2:2891_DCS-MMAE that targets human epidermal growth factor receptor 2 (HER2)-positive breast cancer cells. The conjugate is based on Z_HER2:2891_ affibody and a drug conjugation sequence (DCS), which allowed for site-specific conjugation of the cytotoxic auristatin E molecule (MMAE) to the affibody. We tested the in vitro efficacy of Z_HER2:2891_DCS-MMAE on several parameters, such as cell viability, proliferation, migration, and apoptosis. Our results confirmed that the cytotoxic conjugate efficiently interacts with high affinity with HER2 positive cancer cells, allowing the selective and specific delivery of the cytotoxic payload.

**Abstract:**

A promising approach for the development of high-affinity tumor targeting ADCs is the use of engineered protein drugs, such as affibody molecules, which represent a valuable alternative to monoclonal antibodies (mAbs) in cancer-targeted therapy. We developed a method for a more efficient purification of the Z_HER2:2891_DCS affibody conjugated with the cytotoxic antimitotic agent auristatin E (MMAE), and its efficacy was tested in vitro on cell viability, proliferation, migration, and apoptosis. The effects of Z_HER2:2891_DCS-MMAE were compared with the clinically approved monoclonal antibody trastuzumab (Herceptin^®^). To demonstrate that Z_HER2:2891_DCS-MMAE can selectively target HER2 overexpressing tumor cells, we used three different cell lines: the human adenocarcinoma cell lines SK-BR-3 and ZR-75-1, both overexpressing HER2, and the triple-negative breast cancer cell line MDA-MB-231. MTT assay showed that Z_HER2:2891_DCS-MMAE induces a significant time-dependent toxic effect in SK-BR-3 cells. A 30% reduction of cell viability was already found after 10 min exposure at a concentration of 7 nM (IC_50_ of 80.2 nM). On the contrary, MDA-MB-231 cells, which express basal levels of HER2, were not affected by the conjugate. The cytotoxic effect of the Z_HER2:2891_DCS-MMAE was confirmed by measuring apoptosis by flow cytometry. In SK-BR-3 cells, increasing concentrations of conjugated affibody induced cell death starting from 10 min of treatment, with the strongest effect observed after 48 h. Overall, these results demonstrate that the ADC, formed by the anti-HER2 affibody conjugated to monomethyl auristatin E, efficiently interacts with high affinity with HER2 positive cancer cells in vitro, allowing the selective and specific delivery of the cytotoxic payload.

## 1. Introduction

The human epidermal growth factor receptor 2 (HER2) is a tyrosine kinase receptor that belongs to the family of the epidermal growth factor receptors (EGFRs).

Amplification of HER2 gene is observed in 20–30% of human cancers, especially breast and ovarian cancers [1], and in about 30% of feline mammary carcinomas (FMCs) [2], while its overexpression is correlated with poor prognosis and worse clinical outcomes [3]. The overexpression of HER2 in tumor cells leads to the activation of various signaling pathways involved in cellular proliferation, migration, and apoptosis suppression [4]. Thus, HER2 represents an important pharmacological target for HER2-positive breast cancer therapy. The most used drug targeting HER2 is trastuzumab (Herceptin^®^). This is a humanized IgG1 monoclonal antibody (mAb) that binds to the extracellular domain of the human HER2 protein and is currently used in patients with metastatic breast or gastric cancer characterized by HER2 overexpression [5]. Trastuzumab seems to exert its therapeutic effect through different mechanisms, including the activation of antibody-dependent cellular cytotoxicity [6], the inhibition of the MAPK and PI3K/AKT pathways [7], leading to cell cycle arrest, and by blocking the shedding of the HER2 extracellular domain [8].

Trastuzumab, when combined with chemotherapy, improves overall survival in patients with HER2-positive breast cancer [9]. However, the clinical efficacy of trastuzumab is limited, due to the development of resistance to the drug in a significant number of women with HER2 overexpressing tumors [10].

Target therapy using specific antibodies conjugated with a cytotoxic drug represents an innovative strategy in cancer treatment and a valid alternative to naked antibody-targeted therapy [11]. Antibody-drug conjugates (ADCs) combine the highly specific targeting of mAbs with the potent cytotoxic activity of small molecule agents. The Food and Drug Administration (FDA) approved two ADCs, brentuximab vedotin (Adcetris^®^) and trastuzumab emtansine (Kadcyla^®^), for the treatment of patients with Hodgkin lymphoma and HER2 metastatic breast cancer, respectively [12]. Brentuximab vedotin consists of an anti-CD30 antibody linked to the potent antimitotic drug monomethyl auristatin E (MMAE) [13], whereas trastuzumab emtansine combines trastuzumab with another antimitotic cytotoxic agent, derivative of maytansine (DM1), via a chemical linker [14]. Interestingly, a recent paper described the use of anti-HER2 mAbs and ADCs as a new targeted therapy for feline FMC [15]. Although several ADCs are currently used in clinics, they show some limitations due to their large size (mAbs are 150 kDa and over), which reduces their ability to penetrate solid tumors, and associated high production cost [16].

To overcome these limitations, a new class of affinity ligands based on non-antibody scaffolds has become an attractive alternative to mAbs, due to their smaller size of ~6.5 kDa compared to whole antibodies or antibody fragments (~20–150 kDa), a rapid blood clearance that allows for faster penetration and distribution into tissues, and cost-efficient production in prokaryotic hosts (*Escherichia coli*), in contrast to mAbs that are mainly produced in mammalian cells [17].

These small molecules called affibodies are derived from mutagenesis at specific amino acid residues of the B domain of staphylococcal protein A to increase their chemical stability. The resulting engineered variant is called the Z domain [18]. This Z domain consists of 58 amino acids, and 13 of these surface amino acid residues were randomized to generate affibody libraries, followed by phage display selection against different target proteins, including HER2, EGFR, and amyloid-β peptide [19,20].

In the present study, we developed a rapid and simple method for Z_HER2:2891_DCS purification that, in contrast to the previously described procedure [21], does not require protein tagging. According to previous studies, mAbs conjugated with MMAE show selective antitumor activity in patients with solid tumors [22]. For this reason, we decided to conjugate our affibody to auristatin E, a synthetic analogue of the natural product dolastatin 10 that acts by inhibiting cell division and blocking the polymerization of tubulin [23,24]. MMAE is extremely cytotoxic and is not tumor-specific, for these reasons it cannot be used as a drug itself. However, it is clinically used as payload in ADCs such as brentuximab vedotin and polatuzumab vedotin-piiq [13,25]. Abdollahpour-Alitappeh et al. and Sochaj-Gregorczyk et al. demonstrated by MTT and Alamar Blue assay that free MMAE is cytotoxic in several breast and kidney cancer cell lines tested, with an IC_50_ value in the nanomolar range [21,26].

In a previous study, Sochaj-Gregorczyk et al. used an affinity chromatography approach for the purification of the anti-HER2 affibody Z_HER2:2891_DCS, since Z_HER2:2891_DCS was tagged with GST (Z_HER2:2891_DCS-GST) [21]. However, this purification method gave only 1–5 mg of protein from 1-litre culture. Therefore, we set up another, more efficient and faster method to purify Z_HER2:2891_DCS and then characterized its in vitro binding to HER2 and how it might affect cancer cells.

## 2. Materials and Methods

### 2.1. Generation of the pDEST15-GST Removed-Z_HER2:2891_-DCS

To remove the sequence that encodes for the GST tag from the pDEST15- Z_HER22891_DCS construct, inverse PCR with 5′-phosphorylated primers (Table 1) was performed.

Subsequently, the PCR product was subjected to ligation using T4 DNA ligase (Thermo Fisher Scientific). The resulting construct pDEST-GST removed-Z_HER2:2891_DCS was verified by sequencing (LGC Genomics), which confirmed that the sequence encoding GST had been removed.

### 2.2. Affibody Expression

pDEST-GSTremoved-Z_HER2:2891_-DCS was expressed in an *E. coli* BL21(DE3) pLysS strain. *E. coli* were grown on Luria Bertani (LB) medium supplemented with 100 µg/mL ampicillin and 100 µg/mL of chloramphenicol. The expression of Z_HER2:2891_DCS affibody was induced by the addition of 0.5 mM Isopropyl β-D-1-thiogalactopyranoside. Cells were incubated at 37 °C for 4 h and then harvested.

### 2.3. Affibody Purification

Bacterial pellets were resuspended in an ion exchange buffer (50 mM HEPES buffer, pH 8.1) and sonicated to disrupt the cells. Following centrifugation (50,000× *g*, 1 h, 4 °C) and filtration with a 22 µm syringe filter unit, cell lysate containing untagged Z_HER2:2891_DCS was subjected to ion exchange chromatography. The chromatographic separation was performed using an ÄKTA chromatography system (GE Healthcare, Chicago, IL, USA) with a weak cation exchanger column, HiTrap CM Fast Flow (GE Healthcare).

The elution of the affibody was performed by grading the salt concentration (from 10 mM to1 M NaCl). Then the HEPES buffer pH 8.1 was exchanged to the conjugation buffer (25 mM phosphate, 150 mM NaCl, 0.5 mM EDTA pH 6.8) using a HiTrap Desalting, 1 × 5 mL column (GE Healthcare) prepacked with Sephadex G-25 Superfine. Aliquots of affibody were stored at −80 °C.

### 2.4. MMAE Conjugation Reaction and Z_HER2:2891_DCS-MMAE Purification

MMAE conjugation reaction was performed according to the method described by Sochaj-Gregorczyk et al. [21]. Briefly, for the conjugation reaction, a 30 mM solution of non-reduced ZHER2:2891-DCS in 25 mM sodium phosphate, 150 mM NaCl, and 0.5 mM EDTA pH 6.8 was incubated with 2.5 M excess of MC-Val-Cit-PABC-MMAE (maleimidocaprylvaline-citruline-p-amino-benzyloxycarbonyl-monomethyl auristatin E) (ChiroBlock Gmb) for 12 h at room temperature.

After the conjugation reaction, the mixture was purified by hydrophobic interaction chromatography high-performance liquid chromatography (HIC-HPLC) using an Agilent Eclipse XDB-C18 column and an Agilent 1200 HPLC Liquid Chromatography System (Santa Clara, CA, USA). To elute the affibody we used increasing concentrations of acetonitrile from 20% of buffer A (dH_2_O, 0.1% TFA) to 50% buffer B (acetonitrile, 0.1% TFA). The peak containing Z_HER2:2891_DCS-MMAE conjugates was collected and lyophilized.

### 2.5. Reagents

Trastuzumab was purchased from Hoffman-La Roche Ltd. (Basel, Switzerland). MTT (3-(4,5-dimethylthiazol-2-yl)-2,5-diphenyltetrazolium bromide) and the Annexin V-FITC Apoptosis Detection kit were purchased from Sigma-Aldrich (Milan, Italy). McCoy’s 5a medium, Dulbecco’s Modified Eagle’s Medium, Fetal Bovine Serum (FBS), L-glutamine, Penicillin, and streptomycin were purchased from Euroclone (Milan, Italy). iScript gDNA Clear cDNA Synthesis Kit, the iTaq Universal SYBR Green Supermix, and ECL Western Blotting Detection Reagent were purchased from Biorad (Milan, Italy). ErbB2 (HER2) monoclonal antibody (clone 3B5) was purchased from Thermo Fisher Scientific (Milan, Italy) and HRP-labeled goat anti-mouse IgG was purchased from Abcam (Milan, Italy).

### 2.6. Cell Lines

Human breast cancers SK-BR-3 (high HER-expressing cells) and MDA-MB-231 (basal HER-expressing cells) cell lines were purchased from ATCC. The ZR-75-1 cell line (which overexpresses HER2 [27] without gene amplification [28]) was a generous gift from Dr Elisa Caiola, Mario Negri Institute, Milan. SK-BR-3 cell line (ATCC HTB-30) was cultured in McCoy’s 5a medium (Euroclone) supplemented with 10% Fetal Bovine Serum (FBS). ZR-75-1 cells (ATCC CRL-1500) were cultivated in RPMI-1640 medium (Euroclone) supplemented with 10% FBS. MDA-MB-231 cell line (ATCC HTB-26) was grown in Dulbecco’s Modified Eagle’s Medium (DMEM; Euroclone) supplemented with 10% FBS.

Cells were cultured in 100 mm dishes at 37 °C in a humidified atmosphere containing 5% CO_2_, and when they reached confluence, cells were passaged using a Trypsin-EDTA solution.

### 2.7. Cell Viability Assay

Cells were seeded in 24 well-plates at a density of 1 × 10^5^ cells/well and 7 × 10^4^ cells/well, respectively, and then left to grow for 24 h at 37 °C. At confluency, cells were treated with increasing concentrations of trastuzumab and Z_HER2:2891_DCS-MMAE. Z_HER2:2891_DCS not conjugated with MMAE was used as a negative control.

Cell viability upon treatments was evaluated by the MTT method. IC_50_ values were calculated using GraphPad Prism software (GraphPad Prism software, San Diego, CA, USA).

### 2.8. Cell Proliferation Assay

Cells were seeded in 24-well plates at a density of 2 × 10^6^ cells/well. After 24 h, media were removed, and cells were incubated for 72 h with medium containing 0.4% FBS to synchronize cells at G_0_ phase of the cell cycle. After 72 h, control dishes were counted with a Coulter Counter (Beckman Coulter, Life Scientific, Milan, Italy) and this was considered the “basal” number of cells at T0. Consequently, cells were treated with 5-100-500 nM and 1.25 µM of trastuzumab and 5–100 and 500 nM of Z_HER2:2891_DCS-MMAE or Z_HER2:2891_DCS not conjugated with MMAE in medium supplemented with 10% of FBS for 24, 48, and 96 h. Cells number were measured and compared to the zero time-point.

### 2.9. In Vitro Directional Migration (Wound Healing Assay)

Cells were plated in 24-well plates and grown to confluence. Cell monolayers were scratched with a 200 µL pipet tip in a straight line. Thereafter, the cell monolayer was washed with growth medium to remove detached cells. Cells were then incubated with medium containing 0.4% FBS and 5, 100, and 500 nM of Z_HER2:2891_DCS-MMAE or 5-100-500 nM and 1.25 µM of trastuzumab. Images of the wounded area were taken at the same spot at different time points (0, 6, 24, 48, and 72 h) using an inverted microscope (Axiovert 200; Carl Zeiss, 10× objective lens) equipped with a digital camera. Quantification of the wound area was performed using ImageJ, and cell migration was expressed as a percentage of wound areas at different time-points compared to initial wound area (T0).

### 2.10. Apoptosis Analysis

Cell apoptosis was performed using an Annexin V-FITC Apoptosis Detection kit (Sigma-Aldrich) according to the manufacturer instructions. Briefly, SK-BR-3 and MDA-MB-231 were seeded in 24-well plates and grown to confluence. Cells were then treated for 10 min followed by drug removal and an additional 48 h of incubation in medium alone or for 48 h of continuous exposure to Z_HER2:2891_DCS-MMAE (5–500 nM) and trastuzumab (5 Nm–1.25 µM). Next, cells were washed with PBS, resuspended in 300 µL of binding buffer (25 mM CaCl_2_, 1.4 M NaCl, and 100 mM HEPES/NaOH, pH 7.5), and incubated in the dark for 10 min with 5 µL of Annexin V-FITC conjugate and 10 µL of Propidium Iodide (PI). The percentage of apoptotic cells was evaluated with a flow cytometer (ACEA Biosciences NovoCyte, San Diego, CA, USA)

### 2.11. RNA Isolation and qRT-PCR

SK-BR-3 and MDA-MB-231 were seeded in 24-well/plates at a density of 1 × 10^5^ cells/well and 7 × 10^4^ cells/well, respectively. After 24 h, cells were treated with 5, 100, and 500 nM of Z_HER2:2891_DCS-MMAE and incubated for 24, 48, and 96 h. At each time point, cells were washed once with PBS and RNA extraction was performed using a Direct-zol RNA MiniPrep Plus kit (Zymo Research, Milan, Italy). cDNA was generated by reverse transcription of RNA with an iScript gDNA Clear cDNA Synthesis Kit (Biorad). qRT-PCR was performed using iTaq Universal SYBR Green Supermix (Biorad). Samples were analyzed with a CFX CONNECT^TM^ Real Time detection System (Biorad). Results were normalized to β actin gene expression and the relative quantification was determined by the 2^−ΔΔCT^ method. The primer sequences are listed in Table 2.

### 2.12. Western Blot Analysis

SK-BR-3 cells were treated with 5, 100, and 500 nM of Z_HER2:2891_DCS-MMAE and incubated at 37 °C for 24, 48, and 96 h, while MDA-MB-231 cells were treated with 5, 100, and 500 nM of Z_HER2:2891_DCS-MMAE and incubated for 24 h.

For the preparation of total cell lysates, cells were washed with ice-cold PBS and lysed with lysis buffer (NaCl 150 mM, TRIS 50 mM pH 7.6, NONIDET P-40 0.5% and protease inhibitors (Merck, Milan, Italy)).

Protein concentration was determined using a Pierce BCA Protein Assay Kit (Pierce, Rockford, IL, USA) and samples were run on SDS-PAGE.

HER2 expression was evaluated by using a primary ErbB2 (HER2) monoclonal antibody (3B5) (Thermo Fisher Scientific) diluted 1:1000.

Densitometric analysis was performed using the ImageJ program.

### 2.13. Statistical Analysis

Data are presented as the mean ± SEM of 3 experiments performed in triplicates. Comparison between 2 groups was analyzed by independent t-test and the difference between 3 or more was determined by Dunnett post one-way ANOVA test. Results were considered statistically significant for *p*-values < 0.05.

## 3. Results

### 3.1. Expression and Purification of the Z_HER2:2891_-DCS

In a previous study, since the affibody was fused with a GST tag, the anti-HER2 affibody Z_HER2:2891_-DCS was purified by affinity chromatography followed by size exclusion chromatography [21].

Since this method only allowed us to obtain a very low yield (1–5 mg of affibody from 1 L of bacteria culture), we developed a faster and more efficient method to purify the affibody, after GST tag removal.

**Figure 1:**
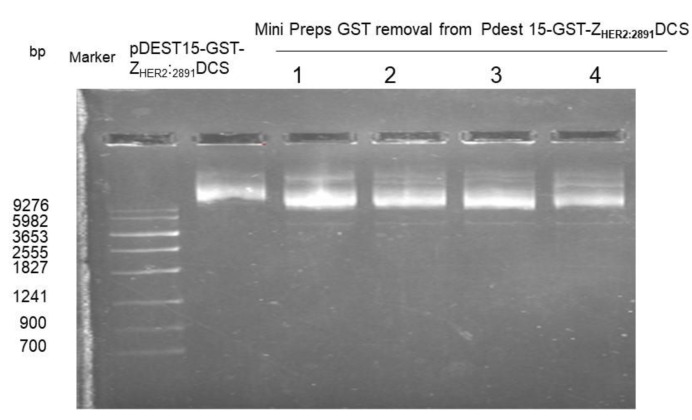
Isolation of DNA plasmid. An agarose gel electrophoresis was used to analyze the four reaction products (1–4) compared to the plasmid with GST.

In order to remove the sequence that encodes GST tag from the pDEST15- Z_HER22891_DCS vector, an inverse PCR with 5′-phosphorylated primers was performed. The removal of the GST tag was confirmed by agarose gel electrophoresis. As shown in Figure 1, we observed a difference of size of the plasmid with GST (used as control) and without GST.

**Figure 2:**
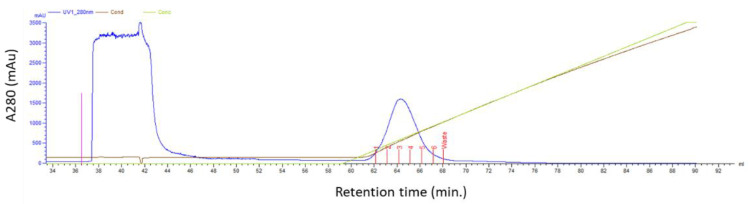
Ion exchange chromatography. The bacterial extract containing high levels of the untagged Z_HER2:2891_DCS was subjected to an ion exchange chromatography (CM sepharose Fast Flow 1 mL column) in HEPES buffer at pH 8.1. The affibody was eluted using a NaCl gradient (see material and methods).

For the purification of the untagged anti-HER2 affibody Z_HER2:2891_DCS, we performed ion exchange chromatography. The 9.38 isoelectric point of our affibody was calculated by using the program EXPASY. We used HEPES buffer pH 8.1, because at this pH, affibody is positively charged and therefore interacts with a cation-exchanger resin, such as the carboxymethyl cellulose, which is negatively charged (Figure 2).

**Figure 3:**
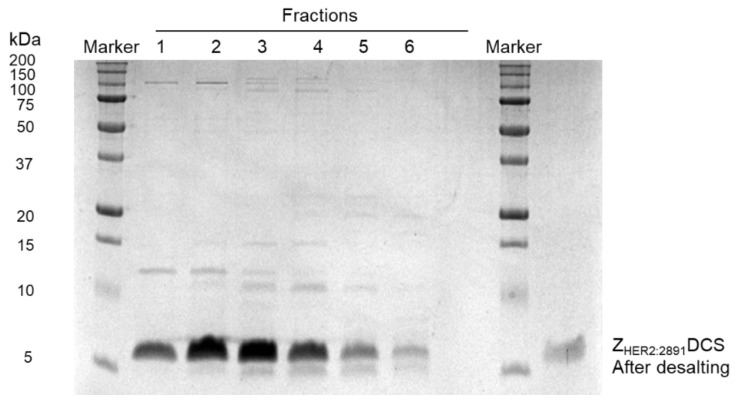
Fraction analysis on SDS-PAGE gel and desalting. All the fractions collected were analyzed on 15% SDS-PAGE gel. The fractions contained the untagged Z_HER2:2891_ were pooled together and subjected to desalting (HiTrap desalting column).

All the fractions collected were analyzed by SDS-PAGE. As we can observe in Figure 3, each fraction contained untagged Z_HER2:2891_DCS. Subsequently, the fractions were pooled together and subjected to desalting (Figure 3) to change the HEPES buffer of pH 8.1 for a phosphate buffer of pH 6.8. We changed pH because the conjugation to MMAE via thiol-maleimide reaction occurs at a pH between 6.8–7.4.

### 3.2. Conjugation of MMAE to Z_HER2:2891_-DCS

Affibody–MMAE conjugation was obtained according to the method described in Sochaj-Gregorczyk et al. [21]. After the conjugation reaction, the mixture was analyzed by HIC-HPLC. This chromatography allowed us to separate unconjugated proteins from the affibody conjugated to the hydrophobic auristatin E. The HPLC chromatogram described by Sochaj-Gregorczyk et al. confirmed the presence of a peak corresponding to Z_HER2:2891_DCS conjugated with MMAE [21].

### 3.3. Z_HER2:2891_DCS-MMAE Affects HER2 Expressing Cell Line Viability In Vitro

The cytotoxicity of Z_HER2:2891_DCS-MMAE was evaluated in HER2-high expressor (SK-BR-3 and ZR-75-1) and low-expressor (MDA-MD-231) cell lines by MTT assay.

Cells were incubated with increasing concentrations (from 1 nM to 500 nM) of Z_HER2:2891_DCS-MMAE and Z_HER2:2891_DCS not conjugated with MMAE, used as negative control, in two different ways: for 10 min followed by drug removal and an additional 48 h of incubation in medium alone, or 48 and 96 h of continuous exposure to the drugs.

**Figure 4:**
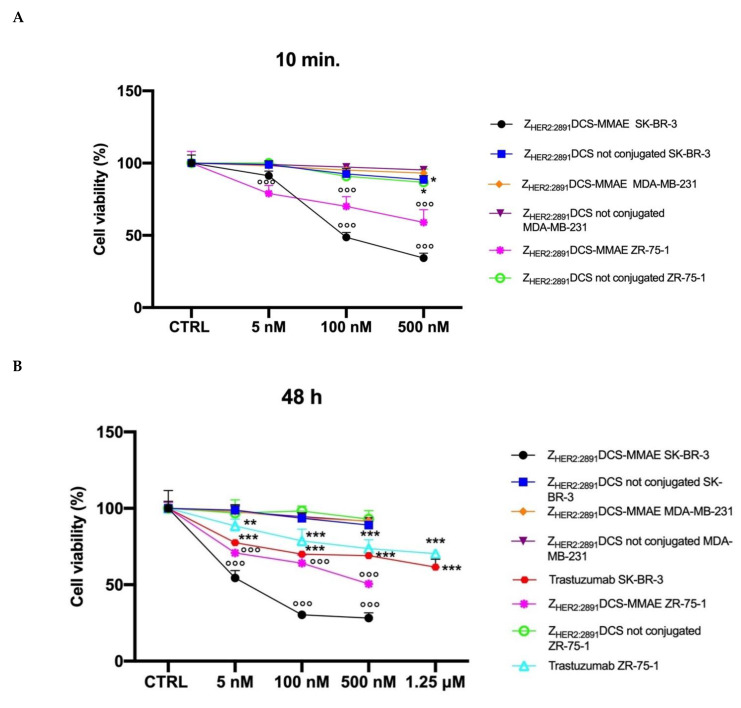

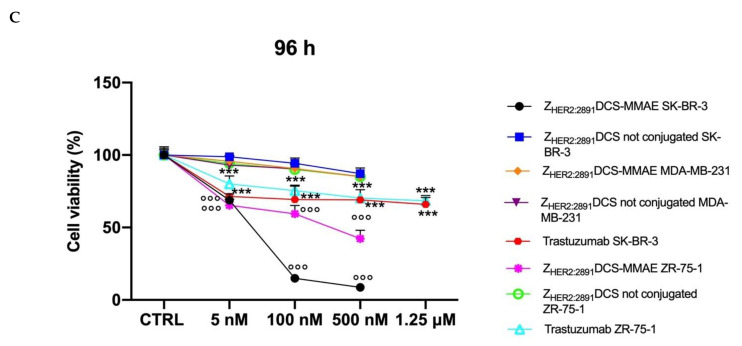
In vitro cell viability assay. SK-BR-3, ZR-75-1 and MDA-MB-231 cells were treated with increasing concentrations (from 5 to 500 nM) of Z_HER2:2891_DCS-MMAE and not conjugated and with increasing concentrations of trastuzumab (from 5 nM to 1.25 µM). The cytotoxicity was measured using an MTT assay after 10 min followed by drug removal and an additional 48 h of incubation in medium alone (Panel **A**) or for 48 and 96 h of continuous exposure to treatments (Panels **B** and **C**, respectively). The *p*-value was determined by one-way ANOVA test with Dunnett post hoc test and considered significant for *p* < 0.05 *, *p* < 0.01 **, *p* < 0.001 *** compared to control and *p* < 0.001 °°° compared to Z_HER2:2891_DCS not conjugated. Data are presented as mean ± SEM.

As shown in Figure 4A, 10 min exposure with Z_HER2:2891_DCS-MMAE was sufficient to reduce cell viability in a concentration-dependent and statistically significant manner in both HER2 expressing cell lines, with an IC_50_ value of 80.2 nM in SK-BR-3 cells.

A stronger effect was observed after 48 h of continuous exposure to Z_HER2:2891_DCS-MMAE, with a 50% reduction of cell viability at a concentration of 5.33 nM (Figure 4B), whereas the longest exposure time (96 h) reduced cell viability close to 0 at a concentration of 500 nM with an IC_50_ of 7.13 nM (Figure 4C). Z_HER2:2891_DCS-MMAE also reduced ZR-75-1 cell viability, although it was less effective (Figure 4A–C) and reached its IC50 of about 500 nM after 48 h of incubation (Figure 4C).

To evaluate if non-conjugated Z_HER2:2891_DCS could affect SK-BR-3 and ZR-75-1cell viability, we treated the cells in the same experimental conditions. As shown in Figure 4A–C, affibody not conjugated to MMAE did not affect cell viability at all time points considered.

As expected, the Z_HER2:2891_DCS-MMAE displayed only a weak in vitro cytotoxic effect on the MDA-MB-231 cells that express a basal level of HER2 at all time points, with a 15% reduction of cell viability only at the highest concentration used, and after 96 h of incubation (Figure 4A–C).

Since trastuzumab is used in patients with HER2-overexpressing metastatic breast cancer, we decided to use it as a reference compound. Therefore, we incubated both SK-BR-3 and ZR-75-1 cells with increasing concentrations of trastuzumab. As shown in Figure 4B,C, at all time points and concentrations tested, trastuzumab showed a lower cytotoxic effect on these cell lines compared to Z_HER2:2891_DCS-MMAE. Of note, not even at the additional higher concentration tested with trastuzumab (1.25 µM) cell viability was reduced up to at least 50%.

### 3.4. Z_HER2:2891_DCS-MMAE Negatively Regulates HER2 Expressing Cell Line Proliferation

Next, we investigated whether treatment with Z_HER2:2891_DCS-MMAE would result in a decreased in vitro proliferation rate of HER2 overexpressing breast cancer cells.

**Figure 5:**
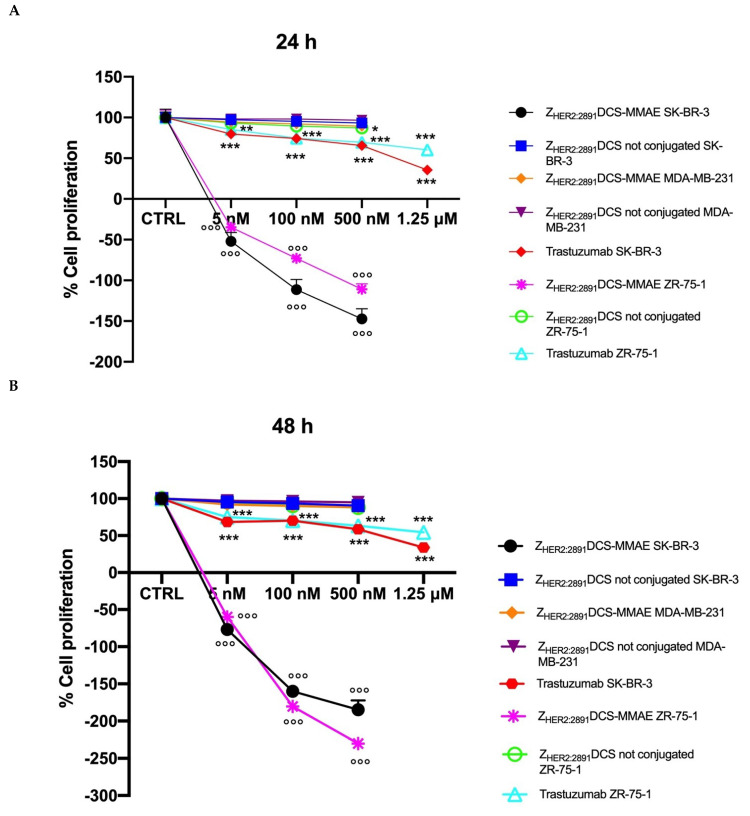

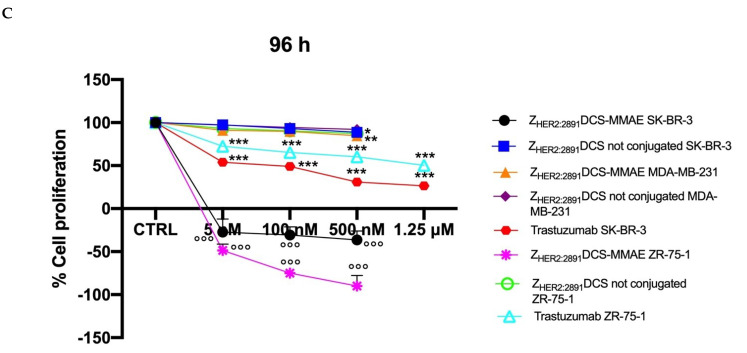
Cell proliferation assay. The effect on cell growth of trastuzumab and Z_HER2:2891_DCS-MMAE and not conjugated, was tested in SK-BR-3, ZR-75-1 and MDA-MB-231 cells. Cells were treated with the substances for 24, 48, and 96 h (Panels **A**, **B,** and **C**, respectively). Cell number was measured with a Coulter Counter. The *p*-value was determined by one-way ANOVA test with Dunnett post hoc test and considered significant for *p* < 0.05 *, *p* < 0.01 ** *p* < 0.001 *** compared to control and *p* < 0.001°°° compared to Z_HER2:2891_DCS not conjugated. Data are presented as mean ± SEM.

As shown in Figure 5, Z_HER2:2891_DCS-MMAE induced a concentration and time-dependent inhibition of cell proliferation in SK-BR-3 and ZR-75-1 cell lines, already having a cytotoxic effect (indicated by a reduction in cell number below the starting basal cell number at time 0 (T0)) after 24 h of treatment, with the strongest effect observed after 48 h. Interestingly, the Z_HER2:2891_DCS-MMAE response was less evident after 96 h of treatment in both HER2 overexpressing cell lines (Figure 4, panels C versus B).

As expected, in the same experimental conditions Z_HER2:2891_DCS-MMAE displayed only a weak inhibition of cell proliferation in MDA-MB-231 at every time point and concentration tested (Figure 5, all panels).

As shown in Figure 5 (all panels), trastuzumab significantly inhibited cell in both SK-BR-3 and ZR-75-1 cell lines by more than 50% only at the highest concentration tested (1.25 µM).

### 3.5. Z_HER2:2891_DCS-MMAE Inhibits SK-BR-3 Migration

**Figure 6:**
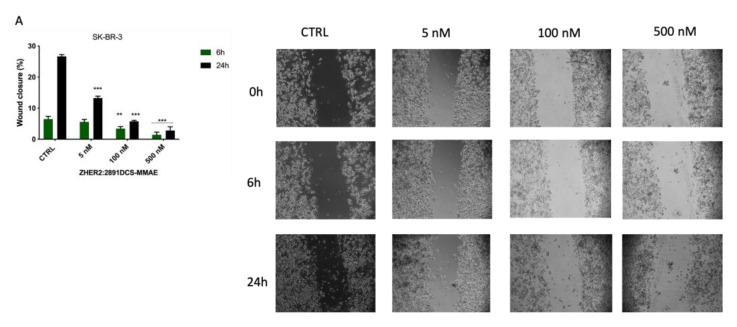

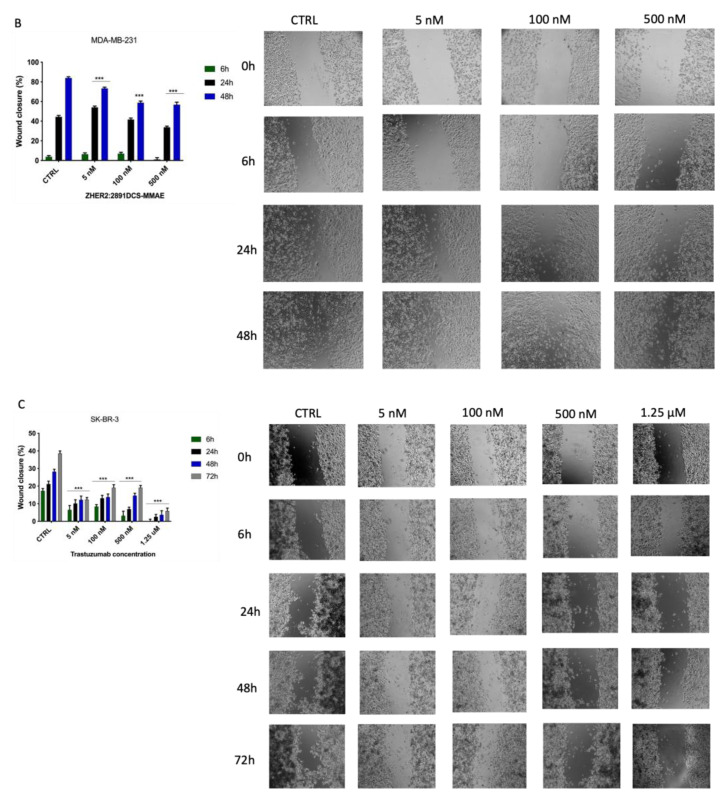
Cell migration assay. The effect on cell migration of Z_HER2:2891_DCS-MMAE and trastuzumab, was tested in SK-BR-3 (**A**,**C**) and MDA-MB-231 cells (**B**) by wound healing assay. Cells were treated with the substances for 6, 24, 48, and 72 h. The *p*-value was determined by one-way ANOVA test with Dunnett post hoc test and considered significant for *p* < 0.01 **, *p* < 0.001 *** compared to control. Data are presented as mean ± SEM.

Next, we evaluated whether Z_HER2:2891_DCS-MMAE treatment would affect SK-BR-3 and MDA-MB-231 cell motility. A low percentage of serum (0.4% of FBS) was used to minimize cell proliferation. The results of the wound healing experiments show that Z_HER2:2891_DCS-MMAE did not significantly affect the wound reclosure after 6 h (Figure 6A). On the contrary, after 24 h of treatment, Z_HER2:2891_DCS-MMAE significantly inhibited SK-BR-3 cell migration in a concentration-dependent manner compared with the untreated cells. After 48 and 72 h of incubation, cells were partially detached from the well surface, which made it impossible to measure the wounded area at these time-points (Data not shown).

In MDA-MB-231 cells, both the untreated group and those treated with Z_HER2:2891_DCS-MMAE showed a similar migration ability, which led to an almost complete reclosure of the wounded area after 48 h of cell incubation (Figure 6B).

As shown in Figure 6C, the migration rate of SK-BR-3 cells was significantly inhibited compared to the control by trastuzumab at all concentrations tested, ranging from 5 nM to 1.25 µM.

### 3.6. Z_HER2:2891_DCS-MMAE Induces Apoptosis of SK-BR-3 Cells

To further investigate the cytotoxic effects of Z_HER2:2891_DCS-MMAE, we next assessed if the compound induced cell death in HER2 positive SK-BR-3 cells. Therefore, we analyzed by flow cytometry the expression of phosphatidylserine, a marker of apoptosis, together with DNA staining, as a readout of cell death due to membrane permeability, using annexin V-FITC and PI, respectively. Treatment with Z_HER2:2891_DCS-MMAE increased the percentage of SK-BR-3 cells undergoing apoptosis (calculated by the sum of cells in early, positive to AnV, and late, double positive for AnV and PI, apoptosis).

**Figure 7:**
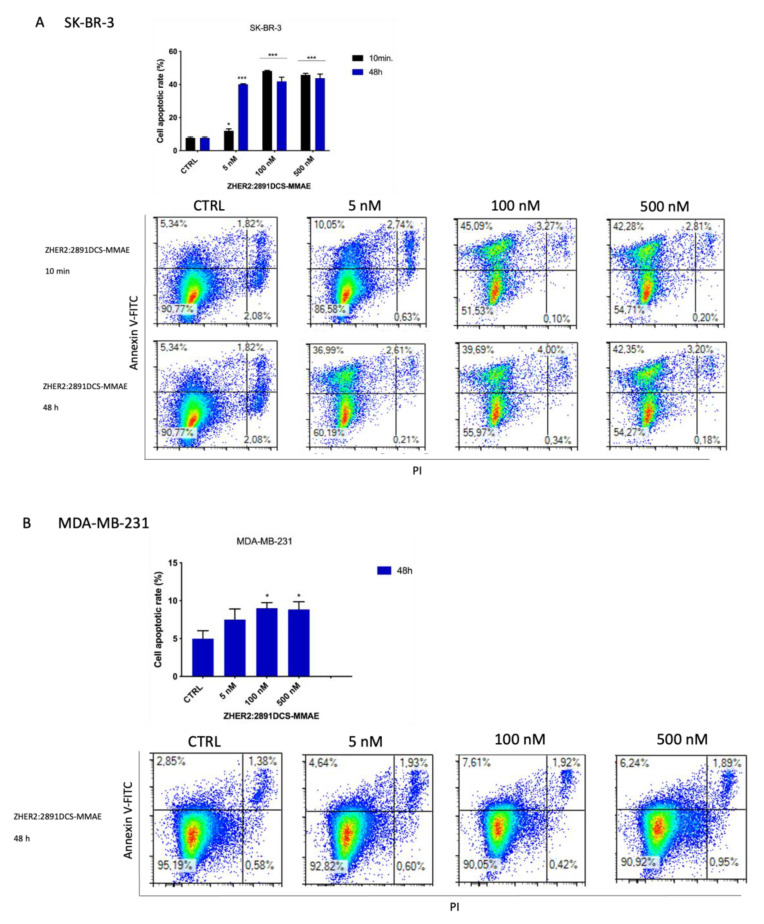

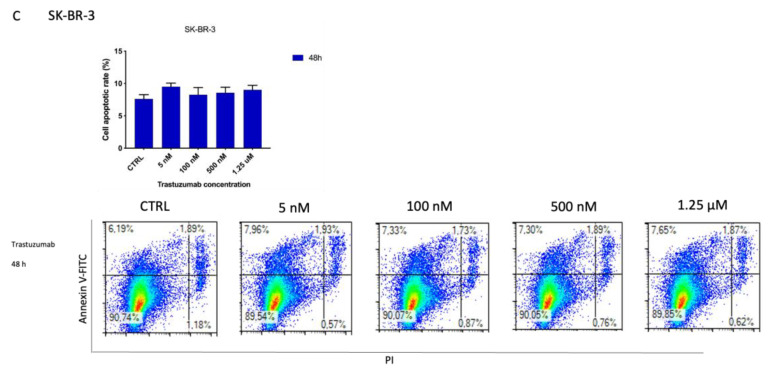
Detection of cell death induced by Z_HER2:2891_DCS-MMAE and trastuzumab treatments. The effect of cellular death induced by Z_HER2:2891_DCS-MMAE and trastuzumab was tested in SK-BR-3 (**A**,**C**) and MDA-MB-231 cells (**B**) by flow cytometry. The top left quadrant represents the percentage of early apoptotic cells (Annexin V^+^/PI^−^), the top right quadrant is the percentage of late apoptotic cells (Annexin V^+^/PI^+^); whereas the bottom left quadrant represents the percentage of live cells (Annexin V^−^/PI^−^) and the bottom right quadrant represents the percentage of necrotic cells (Annexin V^+^/PI^+^). Cells were treated with the substances for 10 min followed by drug removal and an additional 48 h of incubation in medium alone (black bar) or for 48 h of continuous exposure to treatments (blue bar). The *p*-value was determined by one-way ANOVA test with Dunnett post hoc test and considered significant for *p* < 0.05 *, *p* < 0.001 *** compared to the control. Data are presented as mean ± SEM.

After a 10 min exposure followed by drug removal and an additional 48 h of incubation in medium alone, Z_HER2:2891_DCS-MMAE (100 and 500 nM) induced a significant increase (40% each) of apoptotic cells compared to the control. After 48 h of treatment, even the lowest concentration of 5 nM significantly increased the rate of apoptosis, by 40% (Figure 7A). In contrast, after 48 h, no significant effect on apoptosis was observed following trastuzumab treatment in SK-BR-3 cells (Figure 7C).

As expected, 48 h of treatment with Z_HER2:2891_DCS-MMAE did not induce apoptosis in the MDA-MB-231 cell line compared to the control (Figure 7B). Moreover, as shown in the bottom right quadrant of each plot, treatment with Z_HER2:2891_DCS-MMAE or trastuzumab did not induce necrosis in either cell line.

### 3.7. Z_HER2:2891_DCS-MMAE Reduces HER2 Expression by SK-BR-3 Cells

Subsequently, we tested the effects of Z_HER2:2891_DCS-MMAE on the expression of HER2 in SK-BR-3 and MDA-MB-231 cells.

**Figure 8:**
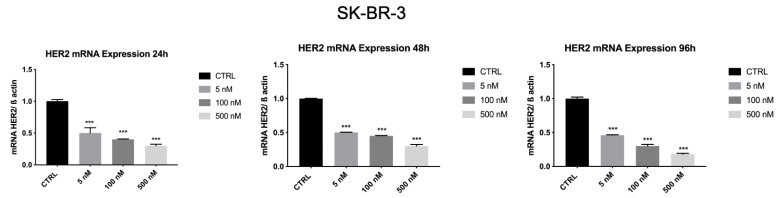
Effect of Z_HER2:2891_DCS-MMAE on HER2 expression in SK-BR-3 cells. Cells were treated for 24, 48, and 96 h. The *p*-value was determined by one-way ANOVA test with Dunnett post hoc test and considered significant for *p* < 0.001 *** compared to control. Data are presented as mean ± SEM.

As shown in Figure 8, HER2 mRNA levels were significantly reduced (*p* < 0.001) by Z_HER2:2891_DCS-MMAE in a concentration and time-dependent manner. The lowest concentration of 5 nM significantly decreased HER2 transcript by 50% within 24 h. The strongest effect was observed after 96 h of treatment, with an 80% inhibition of HER2 mRNA levels.

**Figure 9:**
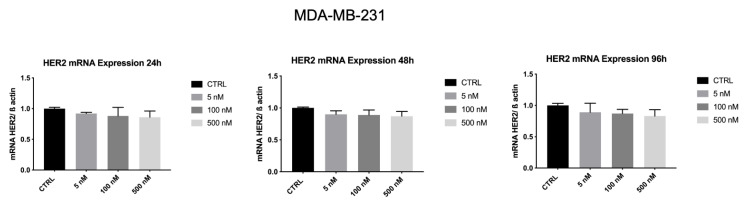
Effect of Z_HER2:2891_DCS-MMAE on HER2 expression in MDA-MB-231cells. Cells were treated for 24, 48, and 96 h. The one-way ANOVA test did not reveal statistically significant differences between the control and treatments. Data are presented as mean ± SEM.

Whereas, as shown in Figure 9, HER2 mRNA levels in MDA-MB-231 were not affected by Z_HER2:2891_DCS-MMAE.

**Figure 10:**
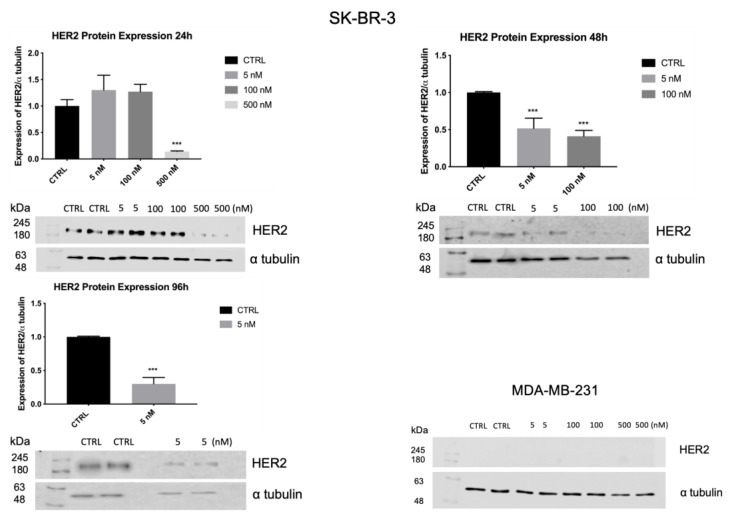
HER2 expression in SK-BR-3 and MDA-MB-231 cell lines. SK-BR-3 were treated for 24, 48, and 96 h, while MDA-MB-231 were treated for 24 h. HER2 expression was measured by Western blot analysis and α tubulin was used as a loading control. The *p*-value was determined by one-way ANOVA test with Dunnett post hoc test and considered significant for *p* < 0.001 *** compared to control. Data are presented as mean ± SEM. The complete western blots are shown in Supplementary Figures S1 and S2.

To confirm the data obtained by RT-PCR, the expression of HER2 in SK-BR-3 cells, was evaluated by Western blot analysis. As shown in Figure 10, after 24 h, the treatment with 5 and 100 nM of Z_HER2:2891_DCS-MMAE did not significantly affect HER2 expression. By contrast, 500 nM of Z_HER2:2891_DCS-MMAE significantly decreased HER2 expression by 90%. The treatment with 100 and 500 nM of Z_HER2:2891_DCS-MMAE after 96 h was cytotoxic and we could not analyze HER2 protein expression.

As expected, MDA-MB-231 does not overexpress HER2.

## 4. Discussion

ADCs represent a successful class of anticancer agents that combine the selectivity of mAbs with the cytotoxic potency of a chemotherapeutic agent [29].

Tissue penetration and biodistribution are important factors, which most of the time seriously limit the response to treatment. One of the major limitations of these molecules is their large size (150 kDa), which limits their ability to penetrate solid tumors [16]. In addition, due to their long serum half-life and slow blood clearance, they are not suitable for radioimmunotherapy or imaging purposes [16]. Another limitation is represented by the fact that many ADCs, including brentuximab vedotin and trastuzumab emtansine, still have a variable drug-to-antibody ratio and variable sites for drug conjugation, thus leading to the formation of heterogeneous species, each with different pharmacokinetic and efficacy profiles [30,31]. Therefore, a promising approach is represented by small carrier proteins able to interact specifically and with a high affinity (in the picomolar to the nanomolar range) with several targets overexpressed in tumor cells such as HER2, EGFR, and IGF-1R [32,33].

Affibody molecules are made of 58 amino acids (with a molecular weight of approximately 6.5 kDa) folded into a three-helical bundle and devoid of cysteines in their structure. Thus, they can be site-specifically modified by introducing one or more cysteine residues into the scaffold, permitting a site-specific conjugation of a cytotoxic payload. In the present work, a DCS that contains a single cysteine residue was introduced at the C-terminus of Z_HER2:2891_, which allowed for site specific conjugation of the cytotoxic MMAE molecule to the affibody via thiol-maleimide chemistry.

Eigenbrot et al. demonstrated that these small molecules have remarkable biophysical properties, such as high thermal stability (Tm = 67 °C), rapid folding, and high solubility in aqueous solutions [34]. The favorable properties of the Z_HER2_ affibody molecule has led to their employment in diagnostic and therapeutic applications.

Affibodies represent a promising approach in terms of imaging because of their rapid biodistribution and rapid blood clearance, due to their small size. Affibodies against several cancer markers, such as HER2, have been developed for tumor imaging [17]. The first radiolabeling of affibody was investigated by Orlova et al. for imaging of HER2 expression. In this study, DOTA-Z_HER2:342_ (ABY-002) was efficiently labelled with indium-111. A biodistribution study of 111In-benzyl-DOTA-Z_HER2:342_ was performed in nude mice bearing LS174T xenografts. In vivo, 111In-benzyl-DOTA-Z_HER2:342_ demonstrated effective tumor uptake 4 h post injection [35].

For therapeutic applications, several groups have developed and characterized affibody molecules interacting with HER2 [36,37]. Zielinski et al. constructed a conjugate based on a modified version of the exotoxin A derived from *Pseudomonas aeruginosa* (PE38) fused to the Z_HER2:342_ and Z_HER2:2891_ affibodies. These constructs efficiently bind and kill cancer cells expressing HER2 after 1 min of exposure [36]. In addition, in vivo studies were carried out using xenograft HER2-overexpressing BT-474, SKOV3, and NCI-N87 tumors. HER2 affitoxin treatment resulted in a 60% volume reduction in BT-474 tumors after the first injection and a significant slowing down of tumor growth in mice bearing SKOV3 and NCI-N87 tumors [36]. Consistent with this, Gräslund et al. investigated the therapeutic efficacy of the Z_HER2:2891_ affibody conjugated with the cytotoxic maytansine derivate mcDM1 in a xenograft of HER2-overexpressing SKOV3 tumors. Treatment with Z_HER2:2891-_ABD-E_3_-mcDM1 led to a significant reduction in tumor size, with a complete tumor regression in some animals at the end of the study [37]. These results demonstrate valuable evidence for the development of an anti-HER2 affibody in cancer-targeted therapy, which represents a promising alternative as a therapeutic agent in clinical practice and also in the veterinary field. In fact, similarly to human breast cancer, FMC is the third most common tumor type in cats. The feline homologue of HER2 is overexpressed in about 30–60% of FMC and is associated with aggressive behavior and poor prognosis [2]. A recently published paper by Ferreira et al. demonstrated that combined treatment with mAbs or an ADC targeting HER2 and the tyrosine kinase inhibitor lapatinib had a synergistic antiproliferative effect in feline cell lines [15].

The aim of our project was to develop a method for the efficient purification and characterization of the Z_HER2:2891_DCS affibody that specifically targets HER2. In contrast to the exotoxin and maytansine derivate used in previous studies, we used MMAE, a potent antimitotic agent that inhibits cell division by blocking the polymerization of tubulin.

In a previous study [21], the Z_HER2:2891_DCS affibody was purified fused with a GST tag. However, this method turned out to be complicated and inefficient. In the present study, the GST tag was removed from the pDEST15-Z_HER2:2891_DCS construct using an inverse PCR, which allowed us to express the affibody without any tag.

Subsequently, Z_HER2:2891_DCS was subjected to an ion exchange chromatography that enabled us to obtain a one-step affibody purification compared to the previously published method, which was carried out in three steps (affinity chromatography, cleavage, gel filtration) [21]. The presently proposed purification method turned out to be simpler and faster, it did not require the removal of the tag, and it was 10 times more efficient than the previous one. The purity of our sample was estimated to be about 90%, and we obtained a much higher yield (25 mg from 1 L of culture).

To gain more insights into the pharmacological effect of Z_HER2:2891_DCS-MMAE, we tested its in vitro effect on tumor cell growth, migration, and apoptosis pathways. As a reference molecule, the clinically approved monoclonal antibody, trastuzumab (Herceptin^®^), was included in the study. Z_HER2:2891_DCS not conjugated with MMAE was used as a negative control.

To assess if Z_HER2:2891_DCS-MMAE can selectively target HER2 overexpressing tumor cells, we used three different cell lines: the human adenocarcinoma cell line SK-BR-3 and ZR-75-1 that express high levels of HER2 (HER2+) [15,27,28,38], and the human mammary gland adenocarcinoma MDA-MB-231, which expresses low (basal) levels of HER2 (HER2-) [39].

The MTT assay showed that Z_HER2:2891_DCS-MMAE had a concentration-dependent and significant toxic effect in both the HER2 overexpressing cell lines, even at the lowest concentration tested of 5 nM (Figure 4). On the contrary, Z_HER2:2891_DCS not conjugated with MMAE did not affect cell viability. The fact that this toxic effect was due to a specific binding to the HER2 was demonstrated by the lack of any significant effects on cell viability in MDA-MB-231 (cells that have only a basal expression of HER2). Due to their small size, affibody molecules have a very short half-life (T_1/2_ < 20 min), since they undergo a rapid renal excretion. As observed by Zielinski et al., the NCI-N87 gastric cell line exposed for 1 min to HER2-affitoxin, followed by an additional 72 h of incubation with medium, resulted in 90% cell death [36]. Our results on cell viability show that exposure to Z_HER2:2891_DCS-MMAE for 10 min, followed by drug removal and an additional 48 h of incubation with medium alone, is sufficient to reduce both SK-BR-3 and ZR-75-1 cell viability by 60% and 40%, respectively, at the highest concentration used.

The cytotoxic effect of Z_HER2:2891_DCS-MMAE was confirmed by measuring cell death by flow cytometry. The total cells undergoing cell death by apoptosis was significantly increased (up to 40%) in SK-BR-3 cells exposed for 10 min to 100 nM of Z_HER2:2891_DCS-MMAE. These findings support the evidence that the high affinity for HER2 receptor allows the affibody to selectively target the cytotoxic payload to HER2 positive cancer cells, thus exerting a cytotoxic activity.

As shown by the MTT assay and cell apoptosis analysis, our reference compound, trastuzumab, displayed only a low cytotoxic effect in SK-BR-3 and ZR-75-1 cells compared to Z_HER2:2891_DCS-MMAE. Consistently, Abdollahpour-Alitappeh et al. showed that different concentrations of trastuzumab (from 1 to 1000 ng/mL) exhibited only a weak cytotoxic effect in SK-BR-3 cells [40].

Since, HER2 overexpression is directly involved in the overstimulation of cell proliferation and migration, we evaluated the in vitro effects of Z_HER2:2891_DCS-MMAE on these parameters. As shown in Figure 5, Z_HER2:2891_DCS-MMAE in SK-BR-3 and ZR-75-1 cells induced cell death starting from 24 h of treatment, with the strongest effect observed after 48 h. Interestingly, the antiproliferative effects of Z_HER2:2891_DCS-MME were less evident after 96 h of treatment, which was probably due to the very short half-life of the affibody molecule. Alternatively, this data might be explained by a reduced cell surface expression of HER2 induced by the affibody, either by stimulating HER2 internalization or by reducing HER2 recycling once internalized.

We next utilized a wound healing assay to evaluate the impact of Z_HER2:2891_DCS-MMAE on cell migration. We found that Z_HER2:2891_DCS-MMAE strongly inhibited SK-BR-3 cell migration, as evidenced by a concentration-dependent decrease in wound area reclosure within 24 h of treatment. Images of the wounded area were also taken after 48 and 72 h (data not shown), but we were not able to measure the lesioned area because of the cell death induced by Z_HER2:2891_DCS-MMAE and consequent cell detachment from the well surface.

In addition, SK-BR-3 treated with increasing concentrations of trastuzumab showed a significant concentration and time-dependent inhibition of cell growth and migration rate. Our results revealed that trastuzumab strongly inhibits cell proliferation, up to 70% at the highest concentration tested, and significantly suppresses SK-BR-3 migration rate compared with untreated cells. Consistently, Emlet et al. reported that trastuzumab, alone and in combination with erlotinib and bevacizumab, exerts a significant growth inhibition on HER2 overexpressing cell lines [41].

Subsequently, we evaluated whether treatment with Z_HER2:2891_DCS-MMAE might affect HER2 expression. Our results demonstrated that, after 24 h of treatment, HER2 mRNA levels were significantly reduced (up to 50%) in SK-BR-3 cells using the lowest concentration of Z_HER2:2891_DCS-MMAE, compared to the control. Furthermore, as shown in Figure 10, 24 h of treatment with 500 nM of Z_HER2:2891_DCS-MMAE drastically decreased HER2 protein expression, by 90%, while after 48 and 96 h the compound was cytotoxic and we could not see any protein on the gel. As expected, the presence of HER2 in MDA-MB-231 was too low to be detected. The fact that Z_HER2:2891_DCS-MMAE can strongly downregulate HER2 expression suggests a potential use of HER2 affibody in the treatment of HER2 positive tumors.

## 5. Conclusions

In conclusion, our experimental data demonstrate that the cytotoxic conjugate formed by the anti HER2 affibody and monomethyl auristatin E efficiently interacts with HER2-expressing cancer cells in vitro, allowing for a selective and specific delivery of the cytotoxic payload. This demonstrates that affibody may be used to target HER2 expressing cells. This technique might allow avoiding some of the problems encountered by using trastuzumab in clinics, such as poor tissue penetration due to its high molecular weight. In addition, the innovative purification procedure applied to isolate the affibody will permit much better yields and reduce production costs.

## Figures and Tables

**Table 1:** List of 5′-phosphorylated primers.

Forward Primer	Reverse Primer
5′ GCCGAAGCCAAATATGCA 3′	5′ CATATGTATATCTCCTTC 3′
3′ CGGCTTCGGTTTATACGT 5′	3′ GTATACATATAGAGGAAG 5′

**Table 2:** List of real-time PCR primer sequences.

Target Gene	Forward Primer	Reverse Primer
βactin	AGTGTGACGTGGACATCCGCA	GCCAGGGCAGTGATCTCCTTCT
HER2	CCAGGACCTGCTGAACTGGT	GTACGAGCCGCACATCC

## Data Availability

Data supporting reported results are available on request.

## Supplementary Materials

The following are available online at https://www.mdpi.com/article/10.3390/biology10080758/s1, Figure S1: representative western blots and densitometry analysis; Figure S2: HER2 expression in MDA-MB-231 cells. Representative western blot of ZHER2:2891DCS-MMAE treatment (5, 100 and 500 nM) after 24 h.
