# Supplementary material for: Purification and In Vitro Evaluation of an Anti-HER2 Affibody-Monomethyl Auristatin E Conjugate in HER2-Positive Cancer Cells
Source: Biology (Basel). 2021 Aug 7;10(8):758. doi: 10.3390/biology10080758 (PMC8389717; doi:10.3390/biology10080758)
Supplement: Supplementary file 1 [file biology-10-00758-s001.zip › biology-1287800-supplementary.pdf]

Article

Purification and In Vitro Evaluation of an Anti-HER2 Affibody-Monomethyl Auristatin E Conjugate in HER2-Positive Cancer Cells

Isabella Damiani, Silvia Castiglioni, Alicja Sochaj-Gregorczyk, Fabrizia Bonacina, Irma Colombo, Valentina Rusconi, Jacek Otlewski, Alberto Corsini and Stefano Bellosta

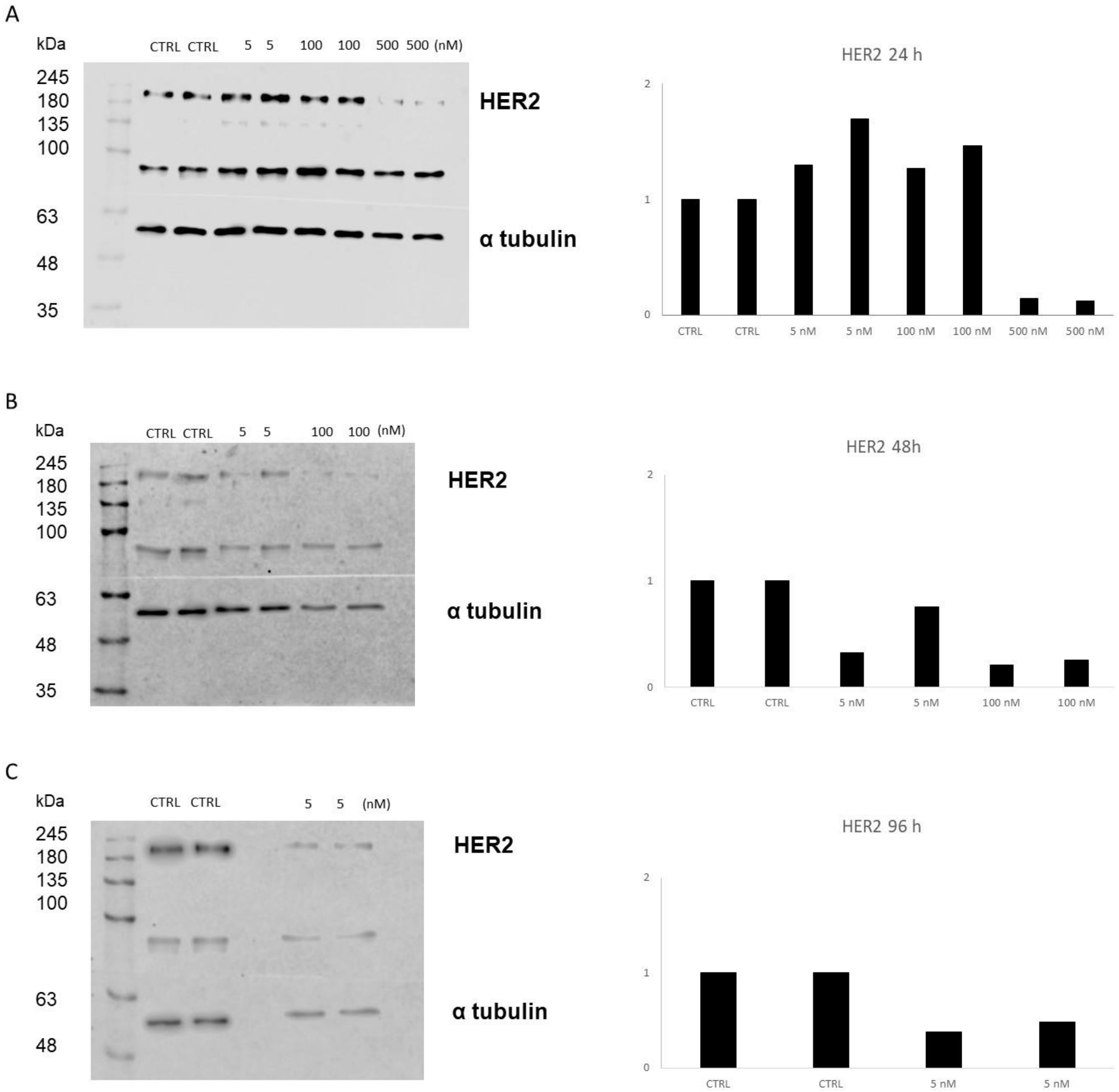

Figure S1. HER2 expression in SK-BR-3 cells. (A) (left) Representative western blot of $Z_{HER2:2891}$DCS-MMAE treatment (5, 100 and 500 nM) after 24 hours. (right) Densitometry analysis of the western blot in A). (B) (left) Representative western blot of $Z_{HER2:2891}$DCS-MMAE treatment (5 and 100 nM) after 48 hours. (right) Densitometry analysis of the western blot in

(B). (C) (left) Representative western blot of Z$_{HER2:2891}$DCS-MMAE treatment (5 nM) after 96 hours. (right) Densitometry analysis of the western blot in (C).

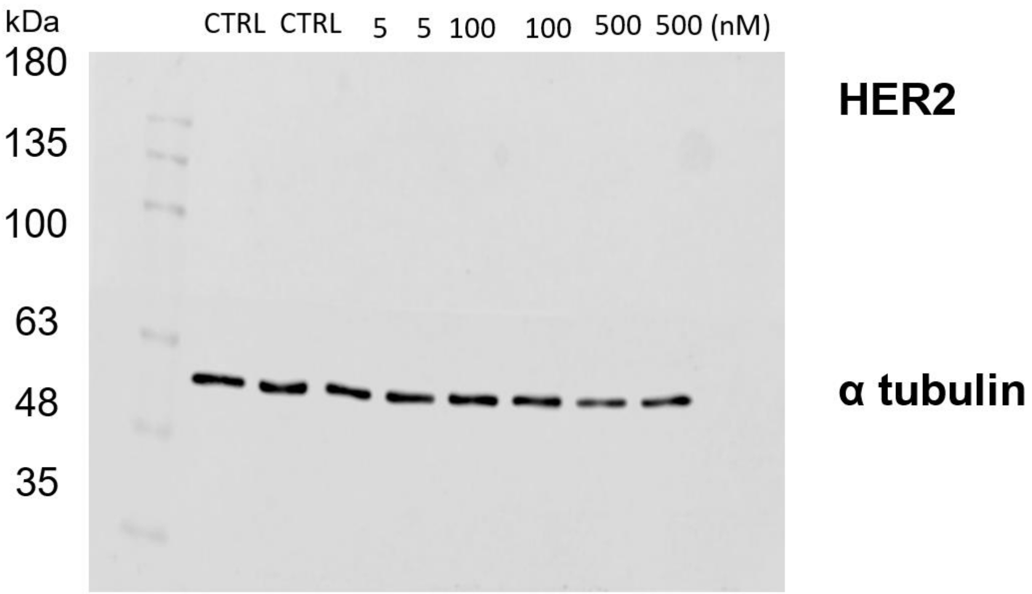

Figure S2. HER2 expression in MDA-MB-231 cells. Representative western blot of Z$_{HER2:2891}$DCS-MMAE treatment (5, 100 and 500 nM) after 24 hours.
